# Effect of acute moderate-intensity cycling on cfDNA levels considering menstrual cycle phases

**DOI:** 10.3389/fspor.2024.1322295

**Published:** 2024-01-29

**Authors:** Akemi Sawai, Takashi Shida, Yoshihiro Hoshikawa, Sho Hatanaka, Mashiro Ueda, Yuri Kato, Katsuyuki Tokinoya, Hiroaki Natsui, Yasushi Kawakami, Kazuhiro Takekoshi

**Affiliations:** ^1^ Research Institute of Physical Fitness, Japan Women’s College of Physical Education, Setagaya City, Japan; ^2^ Department of Clinical Laboratory, Faculty of Medicine, University of Tsukuba, Tsukuba City, Japan; ^3^ Research Team for Promoting Independence and Mental Health, Tokyo Metropolitan Institute for Geriatrics and Gerontology, Itabashi City, Japan; ^4^ Department of Sports Science, Japan Women’s College of Physical Education, Setagaya City, Japan; ^5^ Master’s Program in Medical Sciences, Graduate School of Comprehensive Human Sciences, University of Tsukuba, Tsukuba City, Japan; ^6^ Embodied Wisdom Division, Center for Liberal Education and Learning, Sophia University, Chiyoda City, Japan; ^7^ Department of Sports and Health Science, Japan Women’s College of Physical Education, Setagaya City, Japan

**Keywords:** cfDNA, menstrual cycle, exercise, ovulation, progesterone

## Abstract

**Introduction:**

We aimed to determine the effects of exercise on cell-free DNA (cfDNA) levels and concentration changes during the menstrual cycle in participants with regular menstrual cycles and no exercise habits.

**Methods:**

Eleven sedentary female students with regular menstrual cycles and ovulation performed bicycle exercises at 60% VO_2max_ for 30 min during the menstrual, ovulatory, and luteal phases. Blood samples were collected before (Pre), immediately after (Post 0), 30 min after (Post 30), and 60 min after (Post 60) exercise. Blood concentrations of ovarian hormones, cfDNA, prostaglandin F2a (PGF2α), interleukin-6 (IL-6), and aromatase were evaluated.

**Results:**

Based on the concentration of ovarian hormones, seven individuals were finally analyzed. No significant phase difference was observed in cfDNA across all time points. cfDNA (menstrual phase: *p* = 0.028, ovulatory phase: *p* = 0.018, and luteal phase: *p* = 0.048) and aromatase concentrations (menstrual phase: *p* = 0.040, ovulatory phase: *p* = 0.039, and luteal phase: *p* = 0.045) significantly increased from Pre to Post 0 in all phases. Serum estradiol (E2) levels were significantly higher in the luteal phase at all time points than in the menstrual phase (Pre: *p* < 0.001, Post 0: *p* < 0.001, Post 30: *p* = 0.005, and Post 60: *p* = 0.011); however, serum progesterone (P4) levels were significantly higher in the luteal phase at all time points than in the menstrual (Pre: *p* < 0.001, Post 0: *p* < 0.001, Post 30: *p* < 0.001, and Post 60: *p* < 0.001) and ovulatory phases (Pre: *p* = 0.005, Post 0: *p* = 0.005, Post 30: *p* = 0.003, and Post 60: *p* = 0.003). E2 levels significantly increased from Pre to Post 0 in the ovulatory and luteal phases, whereas P4 levels increased in the luteal phase. Progesterone to estradiol level ratio (P4/E2) changes from Pre to Post 0 (%baseline) during the luteal phase were significantly negatively correlated (*r* = −0.82, *p* = 0.046) with the changes in cfDNA from Pre to Post 0. Furthermore, the repeated measures correlation between P4/E2 and cfDNA level showed a significant negative correlation in ovulatory and luteal phases.

**Discussion:**

The results indicate that while resting cfDNA levels are unlikely to be affected by a woman's menstrual cycle, the increase in cfDNA after exercise is higher in the ovulatory phase (when only E2 increases) and lower in the luteal phase (when E2 and P4 increase with exercise) compared to that in the menstrual phase (when E2 and P4 are in low levels), suggesting the contribution of increased ovarian hormone levels after exercise.

## 1 Introduction

Recently, people have become more health-and-fitness-conscious, and the number of people engaging in exercise is increasing (1). Thus, monitoring performance, fatigue, and recovery is essential for safe and effective training and exercise therapy. Parameters being monitored include neutrophils and cytokines as indicators of inflammation (2–4), cortisol as an indicator of mental fatigue (5), and creatine kinase and myoglobin as the indicators of muscle damage (6); however, their usefulness has varied. Some responses are specific or delayed, while some parameters are unsuitable as low-intensity indicators (6, 7). Here, we focused on cell-free DNA (cfDNA) as a novel marker with the potential to function as a simple and immediate response indicator.

cfDNA is a mixture of DNA fragments that can be observed not only in diseases, such as cancer and atherosclerosis (8–11), but also after exercise. In recent years, cfDNA has been reported to increase nearly 5-fold in ultramarathons (12), 1.4- to 1.7-fold in eight types of strength training (13), 3-fold in weightlifting training (14), and 2.5- to 5-fold during ergometer loading tests (15–17), including acute exercise. Furthermore, cfDNA may be affected by sex differences. Yuwono et al.*,* reported a cohort study of healthy individuals, and Alghofaili et al., Çayir *et al*., and Jylhävä et al. found plasma cfDNA levels of 12.7 and 10.9 ng/ml (18), 707 ± 252 and 586 ± 586 ng/ml (19), and 0.729 and 0.699 μg/ml (20) in men and women, respectively, with higher levels observed in males than in females, suggesting that sex differences may be involved (21). Although a precise mechanism has not been elucidated, previous studies have stated that cfDNA concentrations were examined only in men to avoid the effects of menstruation (22) and fluctuations in hormone concentrations (13) that occur in female participants. Therefore, examining the dynamics of cfDNA in women due to exercise while considering fluctuations in female hormone levels and associated phases in the menstrual cycle is crucial. To date, only two studies have examined cfDNA concentrations during the menstrual cycle in women; however, no studies have found significant differences in cfDNA concentrations in the resting state during the menstrual cycle (23, 24). Pölcher et al. did not include the luteal phase when P4 concentration increased (23), and Yuwono et al. (24) included smokers and individuals taking low-dose estrogen-progestin combination (LEP) preparations, making it impossible to conclude the relationships within the menstrual cycle in the resting state. As the performance and conditioning in females are often affected by the menstrual cycle (i.e., changes in the secretion of ovarian hormones) (25–28), it is necessary to establish a method to appropriately manage menstruation. Therefore, the relationship between the menstrual cycle and cfDNA, which can be used as a simple indicator, and the effects of differences in ovarian hormone levels before and after exercise must examined. Hence, it is essential to first examine women with normal menstrual cycles who do not exercise to rule out the effects of regular exercise. Prior research indicates significant increases in cfDNA concentrations at lactate thresholds during moderate-intensity aerobic exercise (29). Consequently, the present study examined changes in cfDNA before and after moderate-intensity exercise loading, considering the health of participants without exercise habits.

In this study, it was important to consider various factors to ensure that moderate-intensity exercise provides an appropriate load. Thus, we evaluated IL-6 levels to investigate whether moderate intensity aerobic exercise, such as that reaching 60% VO_2_max, effectively induces an inflammatory response. Furthermore, because our study focused on female hormone levels, we evaluated aromatase, which is an enzyme involved in the synthesis of sex hormones. In addition, we specifically evaluated PGF2α levels, which tend to increase considerably during the menstrual cycle, to investigate differences within the menstrual cycle, considering the possible contribution of endometrium-derived cfDNA. Hence, we aimed to determine (i) the cfDNA levels during the menstrual cycle and (ii) changes in cfDNA concentration during the menstrual cycles associated with exercise in individuals with regular menstrual cycles without exercise habits. The results of this study will clarify the effects of exercise on the menstrual cycle and establish the usefulness of biomarkers in exercise for women.

## 2 Materials and methods

### 2.1 Participants

Eleven female students (20–32 years old) enrolled at universities and graduate schools in Tokyo and Chiba prefecture were included in this study. The inclusion criteria were (i) no exercise habits within the last 6 months, (ii) regular menstrual cycle with ovulation, (iii) no oral contraceptive use, (iv) no smoking habit, and (v) no medical disease, trauma, disorder, or psychiatric disorder (30). In this study, women without a daily exercise habit were recruited to eliminate the effects of habitual moderate to high-intensity exercise. Individuals who did not meet these criteria and those who had problems with the measurements on the day of the test were excluded.

### 2.2 Study design

This observational study was conducted between October 2021 and March 2022. VO_2max_ was measured once on the 7th to 14th day of menstruation, and exercise tests were performed once in each of the three phases of the subsequent menstrual cycle. All measurements were performed indoors. Before starting the study, its purpose, content, and the possibility of withdrawing were explained to the participants, and their informed consent to participate was obtained. This study was approved by the Research Ethics Committee of Japan Women's College of Physical Education (2021-7). All procedures were in accordance with the 1964 Helsinki Declaration and its later amendments.

### 2.3 Study procedure

**Figure 1:**
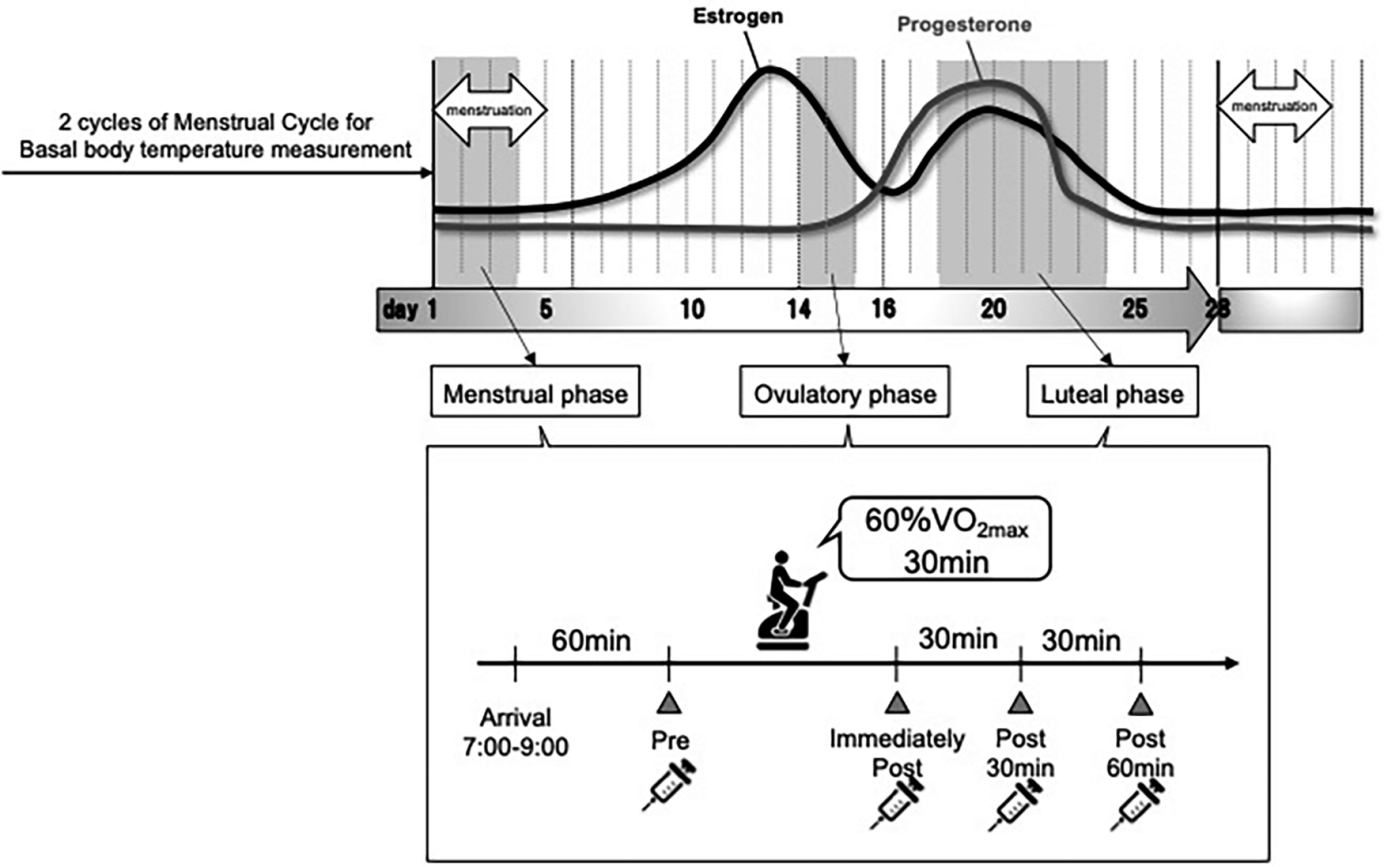
Experimental flow.

The measurement procedure is illustrated in Figure 1. The basal body temperature of participants was measured every morning before and after waking from 2 months before the start of the study until the end. Based on the basal body temperature, menstrual cycle length (number of days), and ovulation detection using an ovulation test, we determined the regular menstrual cycle and schedule for the three exercise tests. For all measurements, alcohol and caffeine intake were prohibited on the day before the test. All exercise tests were performed between 7:00 and 9:00 a.m. On the morning of the exercise test, the body composition was measured prior, expiratory gases and RPE were measured during the exercise test, and venous blood samples were collected before and after the exercise test under fasting conditions.

### 2.4 Phases in the menstrual cycle

The menstrual cycle is divided into three phases: the menstrual phase (a period of menstrual bleeding, low basal body temperature, and low serum E2 and P4 levels), the ovulatory phase (a period of ovulation, high serum E2 levels, and low P4 levels), and the luteal phase (a period after ovulation to the next menstruation, high basal body temperature, and high serum E2 and P4 levels) (31).

In this study, based on the onset of menstruation, basal body temperature, serum hormone levels, and ovulation detected by ovulation testing, the menstrual phase was classified as days 1–4 from menstruation; the ovulatory phase as within 2 days, including the day ovulation was detected; and the luteal phase as 5–10 days before the next expected menstrual onset date.

### 2.5 Measurements

#### 2.5.1 Physical characteristics

The height, weight, body water volume, and fat mass of the study participants were measured. Height was measured using a digital height meter (AD-6400; A&D Company, Tokyo, Japan), while weight, body water volume, and fat mass were measured using a body composition analyzer (InBody770; InBody Co., LTD, Seoul, Korea).

#### 2.5.2 Basal body temperature

Basal body temperature was measured for 30 s every morning before and after awakening by sublingual thermometry using a gynecological thermometer (Terumo electronic thermometer, ET-W525DZ; Terumo Corporation, Tokyo, Japan). Data transfer was performed on the day the participants visited the laboratory for measurement.

#### 2.5.3 Serum ovarian hormone concentration

Ovarian E2 and P4 levels were also measured. After measuring body composition, blood samples (8 ml) were collected from the median vein of the participants in a sitting position at rest immediately, 30 min, and 60 min after the exercise test was performed. Blood was centrifuged at 3,000 rpm for 15 min, and the serum was frozen at −80 °C for storage. Hormone concentrations were measured by chemiluminescent enzyme immunoassay using a mobile immunoluminescent assay device (PATHFAST; LSI Medience Corporation, Tokyo, Japan).

### 2.6 Exercise test and oxygen consumption

An aerobic bike (75XL III; Konami Co., Ltd., Tokyo, Japan) was used to determine the maximal oxygen uptake (VO_2max_) and perform the exercise stress test. The flow meter of the exhaled gas analyzer was calibrated before the start of the experimental day and before each participant's measurement. VO_2max_ was measured at an intensity of 40 W at 60 rpm for 10 min as a warm-up after 3 min of sitting at rest. The exercise was terminated when the pedal speed could no longer be maintained at 50–60 rpm by ramp loading at 15 W/min (the participant was informed when the pedal speed was about to drop below 50 rpm, and after 5 s elapsed after the pedal speed dropped below 50 rpm). Exhaled gases during exercise were measured by the breath-by-breath method using a mass spectrometer for biogas analysis (ARCO-2000; Arcosystem, Chiba, Japan). VO_2max_ was defined as the 1-min average until the maximum oxygen uptake was recorded (VO_2peak_) and divided by the participant's body weight. VO_2max_ was calculated by dividing the VO_2max_ by the body weight of the participant.

In the exercise test during the three periods of the menstrual cycle, after 3 min of sitting at rest on the ergometer, the participants warmed up for 10 min at an intensity of 40 W and 60 rpm. Subsequently, each participant performed a pedaling exercise for 30 min at 60% VO_2max_ intensity. The exhaled gases were measured at rest for 3 min, 2 min after 8–10 min of exercise at 60% VO_2max_, 2 min after 18–20 min, and 2 min after 28–30 min. In this study, we targeted individuals with no regular exercise habits and regular menstrual cycles to eliminate the bias of daily physical activity on parameters such as cfDNA concentration and pro-inflammatory markers. Consequently, the exercise intervention was conducted at a moderate-intensity aerobic level, specifically at 60% VO_2max_, considering the safety of the participants and the intensity at which cfDNA concentrations tend to increase (29).

### 2.7 Cell-free DNA

In this study, plasma samples were used because serum samples may contain cfDNA (32, 33) derived from leukocytes that are dissolved during blood coagulation. After measuring body composition, blood samples (2 ml) were collected from the participant's median vein in a sitting position at rest immediately, 30 min, and 60 min after the exercise test. The cfDNA was extracted using a Plasma/Serum Cell-Free Circulating DNA Purification Mini Kit (Cat. 55100; Norgen Biotek Corp., Thorold, ON, Canada). In total, 40 μl of cfDNA was extracted from 500 μl of plasma according to the manufacturer's protocol. DNA purity was determined using a NanoDrop One Ultra-trace Spectrophotometer (Thermo Fisher Scientific, Waltham, MA, USA).

A High Sensitivity DNA Kit (Cat. 5067-4626; Agilent Technologies, Santa Clara, CA, USA) and a 2100 Bioanalyzer (Agilent Technologies) were used for cfDNA analysis. The analysis was performed in accordance with the manufacturer's instructions. One microliter of the extracted cfDNA sample was used for quantitative analysis.

### 2.8 Sex hormone-synthesizing enzyme

Sex hormones are produced from cholesterol by modifying various sex hormone synthase enzymes (34). E2, a female hormone, is converted from the male hormone testosterone by aromatase (P450arom: aromatase). In this study, we measured blood aromatase concentrations to examine the effects of exercise on cfDNA concentrations and other inflammatory parameters at different levels of female hormones. A Human ARO (aromatase) ELISA Kit (Cat. EH2665; Wuhan Fine Biotech Co., Ltd., Wuhan, China) was used to measure aromatase activity. The assay was performed according to the product protocol, and 100 μl of a 2-fold diluted serum sample was used. The absorbance was measured at 450 nm using a Varioskan LUX Multimode Microplate Reader (Thermo Fisher Scientific). A four-parameter logistic (4PL) curve was generated using ImageJ (NIH, Bethesda, MD, USA) from the absorbance of the standard solution, and the concentration of aromatase in each serum sample was calculated.

### 2.9 Inflammatory response

In parallel with cfDNA, IL-6, and PGF2α levels were measured to confirm the inflammatory response induced by exercise. IL-6 was measured using Human IL-6 Quantikine HS ELISA (Cat.HS600C; R&D Systems, Minneapolis, MN, USA). The assay was performed according to the product protocol, and 100 μl of serum sample was used without dilution. The absorbance was measured at 450 nm using a Varioskan LUX Multimode Microplate Reader (Thermo Fisher Scientific). A 4PL curve was generated from the absorbance of the standard solution using ImageJ, and the level of IL-6 in each serum sample was calculated. PGF2α was measured using a Prostaglandin F2 alpha ELISA Kit (Cat.KA0310; Abnova, Taipei, Taiwan). The manufacturer's instructions were followed, and 100 μl of a 10-fold diluted serum sample was used. The absorbance was measured at 405 nm using a Varioskan LUX Multimode Microplate Reader (Thermo Fisher Scientific). The binding rates of the standard solution and each serum sample were calculated, and a 4PL curve was generated from the binding rates of the standard solution using ImageJ. The PGF2α concentration in each serum sample was calculated.

### 2.10 Statistical analysis

All results of this study are presented as the mean ± standard deviation. Two-way repeated measures analysis of variance (RM-ANOVA) was performed to compare the three different phases over the four time points (Pre, Post 0 min, Post 30 min, and Post 60 min before and after exercise). The Bonferroni multiple testing correction method was used to adjust *p*-values.

The Spearman's rank correlation coefficient was used to examine the relationship between Pre-to-Post 0 changes (%baseline) in the progesterone to estradiol level ratio (P4/E2) and cfDNA, IL-6, aromatase, and PGF2α. Associations between cfDNA and each hormone over time and as well as the associations between %changes in cfDNA and in each hormone were assessed based on the basis of the repeated measurement correlation coefficient.

SPSS ver. 27 (SPSS Inc., Chicago, IL, USA) and R version 4.2.2 (https://www.r-project.org/) with the rstatix, rmcorr, and pwr package (35) were used for statistical analysis. The statistical significance level was set at *p *< 0.05.

## 3 Results

Eleven sedentary female students were enrolled in the study, and seven were included in the final analysis, except for one who withdrew during the experiment and three who showed serum P4 levels <10 ng/ml in the luteal phase.

### 3.1 Basic characteristics

Oxygen uptake significantly increased at 8–10, 18–20, and 28–30 min of exercise compared with that observed in Pre, with values averaging approximately 29–31 ml/min/kg over the three phases. No significant differences were observed between phases at all time points. The mean VO_2max_ was 45.3 ± 3.3 (ml/min/kg).

Table 1 presents the baseline characteristics of the seven participants. No significant differences were observed in the body weight, water, or fat mass during the menstrual, ovulatory, and luteal phases.

**Table 1:** Basic characteristics of participants.

Age (years)	Height (cm)	Weight (kg)	Age at menarche (years)	Menstrual cycle (days)	Menstrual pain (mm)	VO_2max_ (ml/min/kg)	60% VO_2max_ (W)
24.8 ± 1.5	159.0 ± 1.1	52.8 ± 2.0	12.6 ± 0.9	29.9 ± 0.6	28.6 ± 9.3	42.1 ± 3.9	108.1 ± 9.4

**Figure 2:**
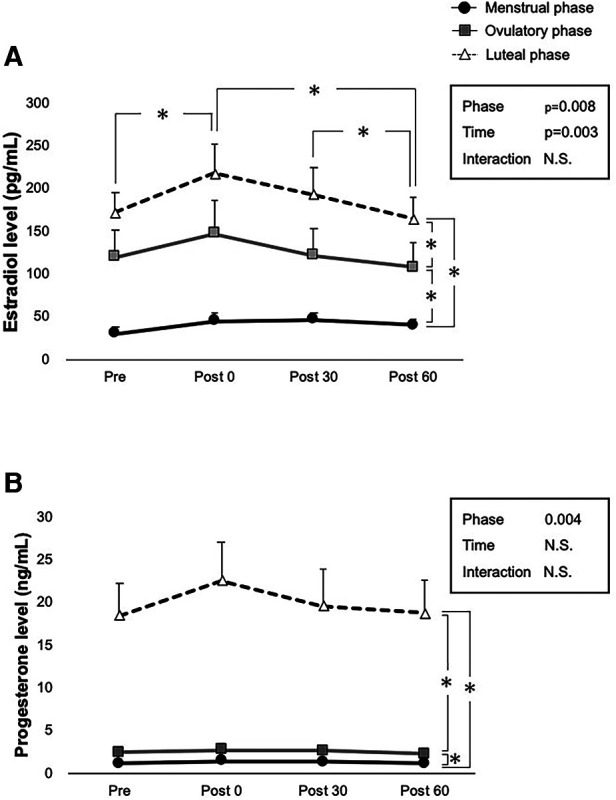
Ovarian hormone levels at each time point over three phases. Interactions of time and phases are evaluated using two-way ANOVA for (**A**) E2 concentration, and (**B**) P4 concentration in pre-exercise (Pre), immediately after exercise (Post 0), 30 min after exercise (Post 30), and 60 min after exercise (Post 60). Menstrual, ovulatory, and luteal phases are represented by black circles with black lines, gray squares with gray lines, and white triangles with dashed lines, respectively. B indicates *p* < 0.05 between time points in the ovulatory phase, and c in the luteal phase. *indicates *p* < 0.05 between time points and phases according to the Bonferroni post-hoc test.

Serum E2 and P4 concentrations before and after exercise are presented in Figures 2A, B. No statistically significant interactions were observed between both hormones. Significant main effects in the phase (*p* = 0.008) and time (*p* = 0.003) were observed for the serum E2 level (Figures 2A). Comparisons made using the pairwise paired *t*-test showed that exercise had a significant incremental effect on E2 levels at Post 0 (*p* = 0.005), and significant decreases were observed after exercise (*p* = 0.002 for Post 60 vs. Post 0, *p* = 0.04 for Post 60 vs. Post 30). Furthermore, comparisons made using pairwise paired *t*-test showed significant differences between the menstrual and ovulatory phases (*p* < 0.001), menstrual and luteal phases (*p* = 0.029), and ovulatory and luteal phases (*p* < 0.001), respectively. Furthermore, the main effect of the phase was significant for serum P4 levels (*p* = 0.004) (Figure 2B). Comparisons made using pairwise paired *t*-test showed significant differences between the menstrual and ovulatory phases (*p* < 0.001), menstrual and luteal phases (*p* < 0.001), and ovulatory and luteal phases (*p* < 0.001), respectively.

### 3.2 cfDNA levels

**Figure 3:**
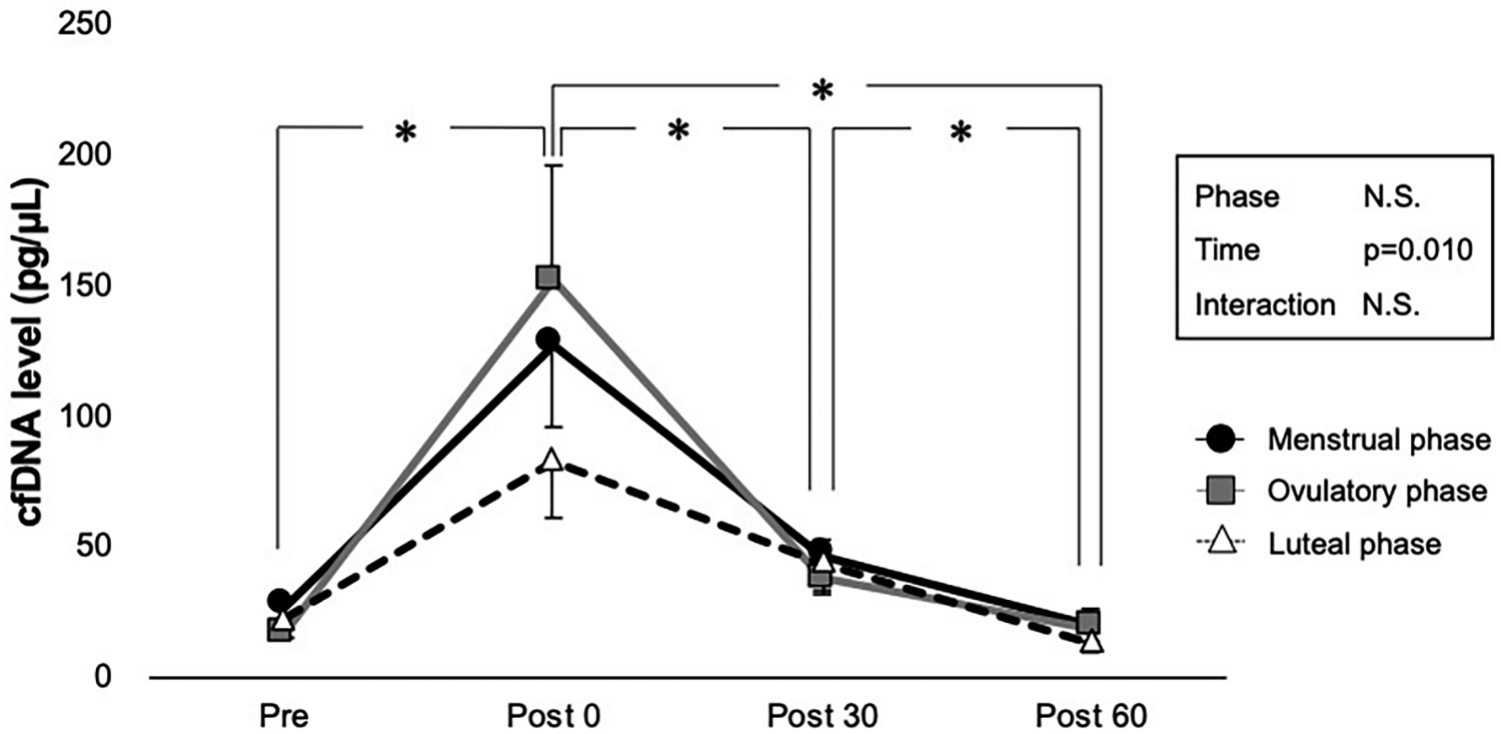
Interaction of time and phases are shown by two-way ANOVA for cfDNA level. Menstrual, ovulatory, and luteal phases are represented in black circles with black lines, gray squares with gray lines, and white triangles with dashed lines, respectively. *Indicates *p* < 0.05 between time points according to the Bonferroni *post hoc* test.

The cfDNA concentrations before and after exercise are shown in Figure 3. No statistically significant interaction was observed between phase and time on cfDNA levels (*p* = 0.327); however, the main effect of time was significant (*p* = 0.010). Comparisons using pairwise paired *t*-test showed that exercise had a significant incremental effect on cfDNA levels at Post 0 (*p* = 0.002) but not at Post 30 (*p* = 0.302) and Post 60 (*p* = 0.954). Significant decreases in cfDNA were observed between all time points after exercise (*p* < 0.001 for Post 30 vs. Post 0, Post 60 vs. Post 0, and Post 60 vs. Post 30). The main effect of phase was not significant (*p* = 0.814).

### 3.3 PGF2α, IL-6, and aromatase levels

**Figure 4:**
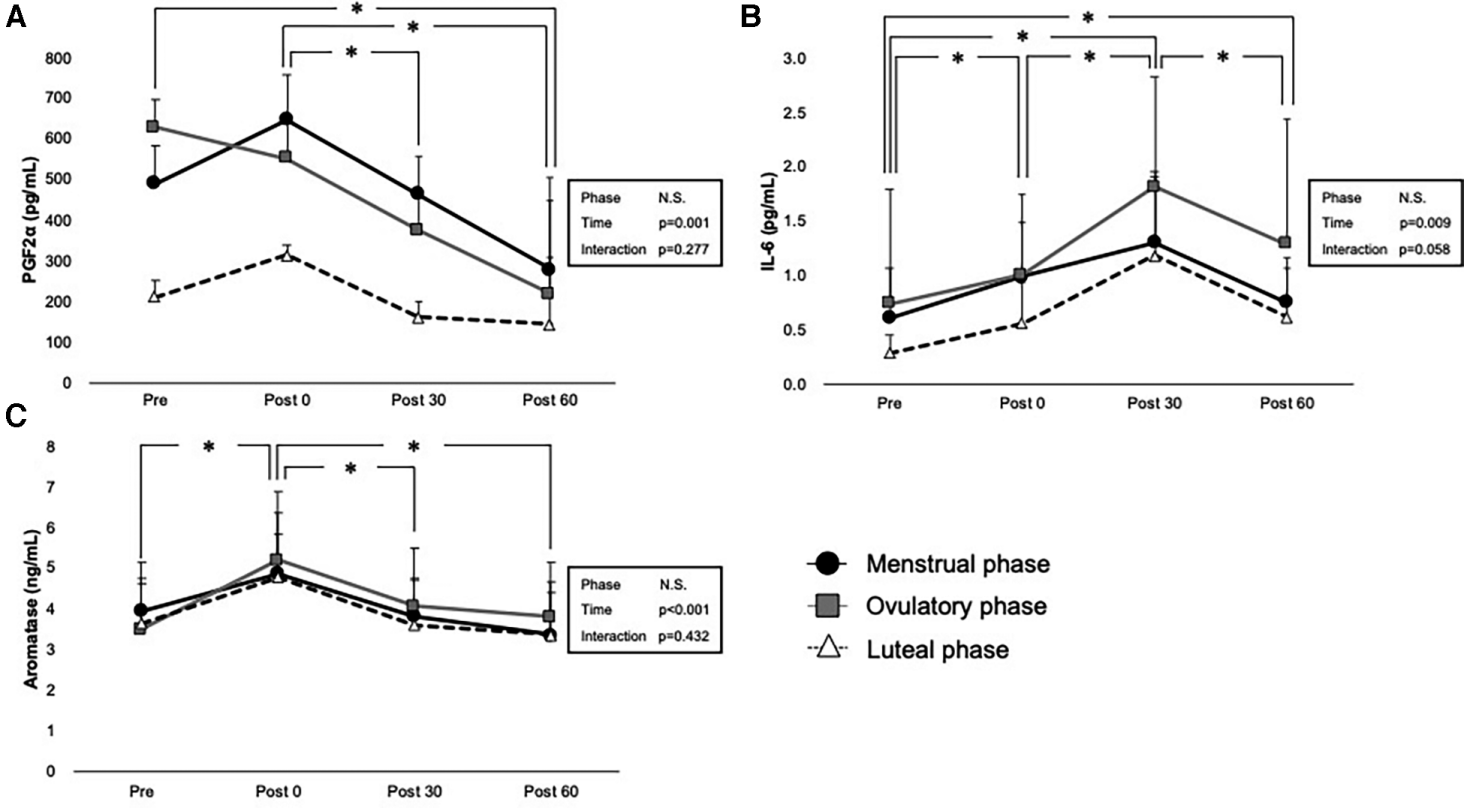
Interaction of time and phases are shown by two-way ANOVA for (**A**) PGF2a, (**B**) IL-6, and (**C**) aromatase levels. Menstrual, ovulatory phase, and luteal phases are represented in black circles with black lines, gray squares with gray lines, and white triangles with dashed lines, respectively. * indicates *p* < 0.05 between time points according to the Bonferroni *post hoc* test.

PGF2α, IL-6, and aromatase levels before and after exercise are shown in Figures 4A–C. No statistically significant interaction was observed between phase and time on these hormone levels (*p* = 0.277 for PGF2α, *p* = 0.058 for IL-6, and *p* = 0.432 for aromatase); however, the main effect of time was significant in each PGF2α (*p* = 0.001), IL-6 (*p* = 0.009), and aromatase (*p* < 0.001). The main effect of phase was not significant (*p* = 0.071 for PGF2α, *p* = 0.324 for IL-6, and *p* = 0.237 for aromatase). Comparisons using pairwise paired *t*-test showed a significant decrease in PGF2α in Pre to Post 60 (*p* = 0.026), Post 0 to Post 30 (*p* = 0.030), and Post 0 to Post 60 (*p* < 0.001).

Exercise had a significant incremental effect on IL-6 levels at Post 0 (*p* = 0.007), Post 30 (*p* < 0.001), and Post 60 (*p* = 0.032), showing the peak level on Post 30 (*p* < 0.001 for Post 0 vs. Post 30, *p* < 0.001 for Post 30 vs. Post 60).

A significant increase in aromatase was observed immediately after exercise (*p* < 0.001 for Pre vs. Post 0), and it significantly decreased after exercise (*p* < 0.001 for Post 0 vs. Post 30) and Post 60 (*p* < 0.001 for Post 0 vs. Post 30).

### 3.4 Correlation with cfDNA

Table 2 shows the repeated measures correlation between cfDNA and each hormone over the three phases, and Table 3 shows the repeated measures correlation between changes in cfDNA and each hormone over the three phases. Table 4 shows the correlation between Pre-to-Post 0 changes (%baseline) in P4/E2 and cfDNA levels over 3 phases.

**Table 2:** Repeated measures of correlation between cfDNA and each hormone over three phases.

	Menstrual phase		Ovulatory phase		Luteal phase	
	*r*_rm_	*p*-value	*r*_rm_	*p*-value	*r*_rm_	*p*-value
P4/E2	0.07	0.757	−0.56	0.007	−0.45	0.038
PGF2α	0.38	0.078	0.15	0.505	0.14	0.545
Interleukin-6	0.20	0.368	0.15	0.494	0.12	0.601
Aromatase	0.52	0.013	0.61	0.002	0.63	0.002

*r*_rm_, repeated measures correlation coefficient.

**Table 3:** Repeated measures correlation between changes (%baseline) in cfDNA and each hormone over three phases.

	Menstrual phase		Ovulatory phase		Luteal phase	
	*r*_rm_	*p*-value	*r*_rm_	*p*-value	*r*_rm_	*p*-value
P4/E2	0.19	0.489	−0.35	0.198	−0.23	0.400
PGF2α	0.28	0.311	0.22	0.425	0.20	0.479
Interleukin-6	−0.07	0.803	−0.12	0.658	0.17	0.540
Aromatase	0.64	0.010	0.60	0.018	0.43	0.111

*r*_rm_, repeated measures correlation coefficient.

**Table 4:** Correlation between Pre-to-post 0 changes (%baseline) in P4/E2 and cfDNA over three phases.

menstrual cycle	Progesterone to estradiol ratio					
	Menstrual phase		Ovulatory phase		Luteal phase	
	*r*	*p*-value	*r*	*p*-value	*r*	*p*-value
cfDNA level (pg/*μ*l)	0.40	0.429	−0.64	0.096	−0.82	0.046

**Figure 5:**
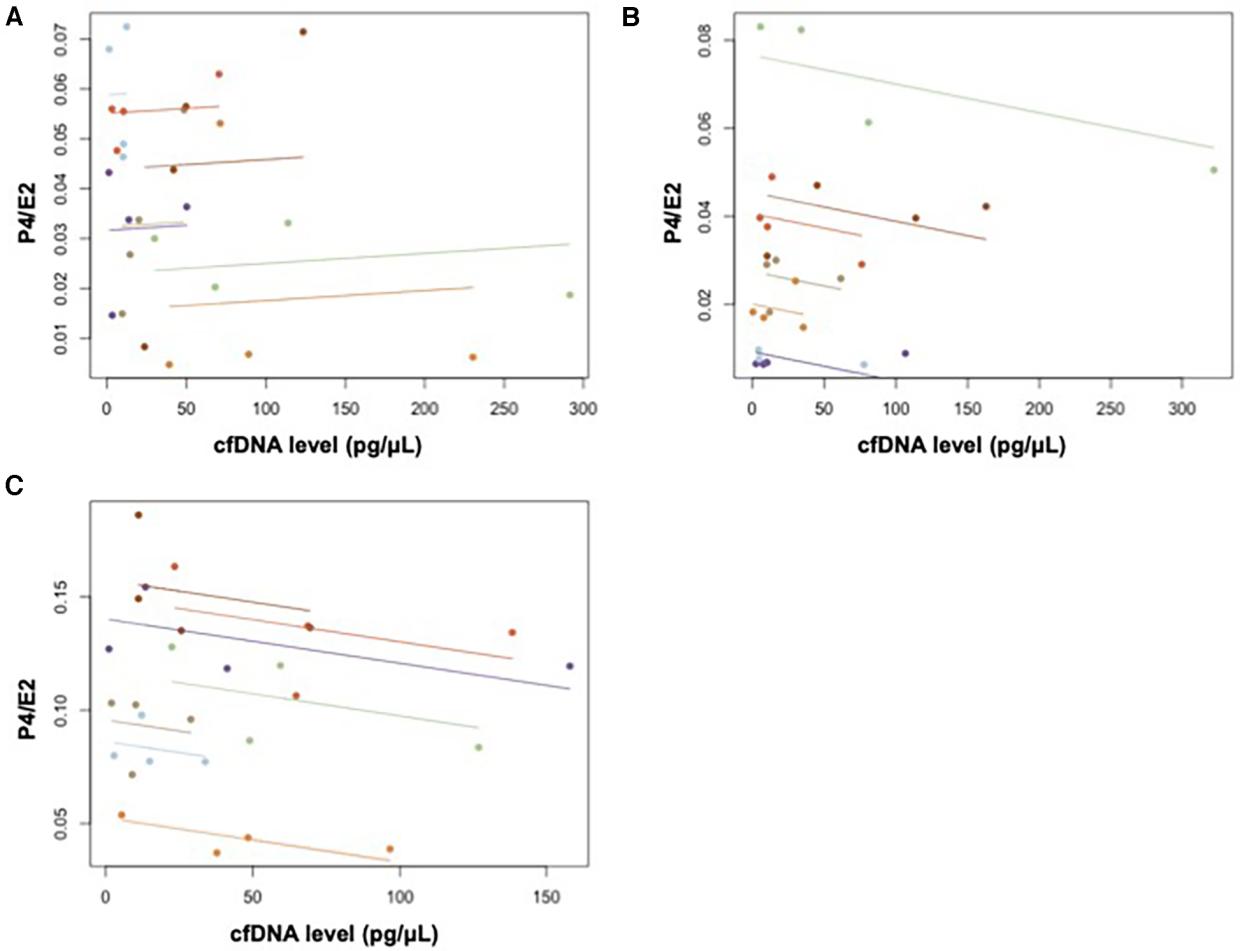
Repeated measures correlation between cfDNA and P4/E2 in (**A**) menstrual, (**B**) ovulatory, and (**C**) luteal phases.

According to Table 2 and Figure 5, the results indicate the association between cfDNA levels and each hormone. The association between P4/E2 and cfDNA was observed in the ovulatory and luteal phases (ovulatory phase; *r*_rm_ = −0.56, *p* = 0.007, statistical power = 0.80, luteal phase; *r*_rm_ = −0.45, *p* = 0.038, statistical power = 0.57); however, no association was found in the menstrual phase (*p* = 0.757, statistical power = 0.06). Moreover, point estimates of the association between PGF2α and cfDNA were higher in the menstrual phase than in the ovulatory and luteal phases. Aromatase was associated with cfDNA in all three phases, showing a significant positive correlation (menstrual phase: *r*_rm_ = 0.52, *p* = 0.013, statistical power = 0.73; ovulatory phase: *r*_rm_ = 0.61, *p* = 0.002, statistical power = 0.89; and luteal phase: *r*_rm_ = 0.63, *p* = 0.002, statistical power = 0.91).

According to Table 3, the results of the association between changes (%baseline) in cfDNA levels and each hormone showed that P4/E2, PGF2a, and IL-6 were not significantly associated with cfDNA over the three phases. However, aromatase was associated with cfDNA in both menstrual and ovulatory phases, showing a significant positive correlation (menstrual phase: *r*_rm_ = 0.64, *p* = 0.010, statistical power = 0.92; ovulatory phase: *r*_rm_ = 0.60, *p* = 0.018, statistical power = 0.87), but not in the luteal phase.

A significant negative correlation was observed between the Pre- to-Post 0%changes in P4/E2 and cfDNA levels in the luteal phase as shown in Table 4 (*r* = −0.82, *p* = 0.046, statistical power = 0.58).

## 4 Discussion

This study investigated the levels of circulating cfDNA before and after exercise during three distinct phases of the menstrual cycle: menstrual (when both ovarian hormone levels are low), ovulatory (characterized by elevated estrogen levels), and luteal phases (when both hormones are at high levels). In this study, we determined the resting cfDNA levels during the menstrual cycle and changes in cfDNA levels during the cycle in individuals with a regular menstrual cycle. Our results showed that the baseline values did not change significantly during the menstrual cycle, and exercise-induced changes in cfDNA levels and related blood markers may be associated with female hormone levels.

The mean VO_2max_ in this study was 45.3 ± 3.3 (ml/min/kg), 60% of which amounts to 27.2 (ml/min/kg). The oxygen uptake results suggest that the exercise load in this study was equivalent to 70% VO_2max_ in this study. This exercise intensity, at 70% of VO_2max_, corresponds to a moderate-intensity exercise. Considering that athletes frequently reach this intensity (36), it can be concluded that a practical intensity was used for the purposes of this study.

No significant main effect of the phase was observed for cfDNA in this study, which is consistent with previous studies showing no significant variation in resting cfDNA levels during the menstrual cycle (23, 24). Nevertheless, Pölcher *et al*. omitted the luteal phase during the documented increase in P4 concentration (23), and Yuwono *et al*. (24) incorporated individuals who were smokers or who used low-dose estrogen-progestin combination (LEP) preparations, rendering it challenging to infer conclusions regarding relationships within the menstrual cycle in a resting state.

In this study, pairwise paired *t*-test comparisons showed that exercise had a significant incremental effect on cfDNA levels at Post 0, indicating the influence of exercise load. cfDNA concentrations reportedly increased up to five-fold from resting levels in an exercise stress test using all-out cycling (16, 17), suggesting that a certain level of exercise stress influenced cfDNA concentrations in the current study.

Aromatase level showed a significant positive correlation with the cfDNA level in the repeated measures correlation across the three phases. The expression of aromatase increases immediately after exercise (37), and aromatase is expressed in skeletal muscle, where it synthesizes estrogen via dehydroepiandrosterone (DHEA) (38, 39). Through repeated measures correlation, a significant negative correlation was observed between P4/E2 and cfDNA in the ovulatory and luteal phases, as well as in the %changes between Pre to Post 0 in the luteal phase. The ovulatory phase is characterized by elevated estradiol levels, while the luteal phase has high levels of both estradiol and progesterone. Therefore, compared to the menstrual phase with low hormone levels, the increase in cfDNA concentration due to exercise may be attributed to the individual effect of estradiol and the combined effects of both hormones.

The repeated measures correlation between PGF2α and cfDNA concentration during the menstrual phase had a higher point estimate than those during the other phases (*r*_rm _= 0.38, *p* = 0.078). None of the study participants reported menstrual symptoms severe enough to interfere with measurements. Notably, a significant increase was only observed during the menstrual phase when compared before and immediately after exercise (*p* = 0.042, data not shown). PGF2α causes menstrual pain, and heightened production of PGF2α has been linked to the heightened sensitivity of uterine muscle fibers, a consequence of impaired blood flow resulting from vigorous contractions of the myometrium (40, 41). Therefore, it can be hypothesized that an increase in cfDNA concentration during menstruation may be observed in women with severe menstrual pain, such as those with dysmenorrhea. Further investigation is necessary to explore this relationship.

The sample size in this study is small (*n* = 7). While this may be a limitation, the assessment of cfDNA secretion before and after exercise during the menstrual, ovulatory, and luteal phases based on the evaluation of female hormone concentrations provides preliminary insights for future studies targeting athletes. These findings are considered the minimum knowledge base for future investigations in this population.

Based on the findings of this study, we propose that future research should focus on individuals who engage in regular exercise or athletes. Even when similar exercises are performed, differences in the dynamics of cfDNA before and after exercise have been reported depending on the presence or absence of exercise habits (42). This may involve the potential influence of psychological stress (29), and elevated cfDNA concentrations can persist for 2–3 days after prolonged exercise loads (43, 44), suggesting potential differences compared to transient exercise. Additionally, our study revealed that high levels of female hormones associated with exercise, as well as the effect of PGF2α on uterine contractions, can impact cfDNA. Therefore, future research must consider the effects of menstrual symptoms and menstrual status (such as oligomenorrhea and amenorrhea). Menstrual irregularities, particularly rare or absent menstruation, are not uncommon among athletes, and these individuals may have a reduced influence from female hormones. As a future prospect, we aim to include individuals with different menstrual statuses to gain a better understanding of the relationship between female hormones and cfDNA dynamics during exercise.

To our knowledge, this study is the first to investigate the levels of circulating cfDNA before and after exercise during three distinct phases of the menstrual cycle: the menstrual (when both ovarian hormone levels are low), ovulatory (characterized by elevated estrogen levels), and luteal phases (when both hormones are at high levels). This research may offer novel insights into exercise physiology in relation to cfDNA, a biomarker that immediately responds to exercise, and its interaction with ovarian hormone concentrations.

## 5 Conclusions

When the female menstrual cycle is considered, resting cfDNA levels are unlikely to be affected. However, the dynamics of cfDNA through acute moderate-intensity exercise may be influenced by the ratio of ovarian hormone concentrations.

## Data Availability

The original contributions presented in the study are included in the article, further inquiries can be directed to the corresponding author/s.
